# Intergenerational Religious Discordance and Relationship Quality Between Older Parents and Adult Children

**DOI:** 10.1093/geroni/igaf122.952

**Published:** 2025-12-31

**Authors:** Merril Silverstein, Woosang Hwang, Maria Brown, Wencheng Zhang

**Affiliations:** Syracuse University, Syracuse, New York, United States; Sookmyung Women’s University, Seoul, Republic of Korea; Syracuse University, Syracuse, New York, United States; Purdue University, West Lafayette, Indiana, United States

## Abstract

In this analysis we examined intergenerational religious discordance and its association with emotional closeness, conflict, frequency of contact, and emotional support exchanged between older parents and their adult children. We considered discordance as absolute difference scores in four dimensions of religiosity: denomination, intensity, frequency of service attendance, and belief in God. Data from the Longitudinal Study of Generations collected in 2016 and 2022 were used in four sub-samples of dyads: 157 mother-son, 180 mother-daughter, 124 father-son, and 113 father-daughter dyads. The results of this analysis will pinpoint whether adverse impacts of religious differences between older parents and their middle-aged adult children have their basis in denominational identification, congregational practice, self-concept, or deistic beliefs. A sub-analysis focuses on whether children have more strained relationships with their parents if they married outside their parents’ religious denomination.

